# Supplier-dependent differences in intermittent voluntary alcohol intake and response to naltrexone in Wistar rats

**DOI:** 10.3389/fnins.2015.00424

**Published:** 2015-11-05

**Authors:** Shima Momeni, Lova Segerström, Erika Roman

**Affiliations:** Department of Pharmaceutical Biosciences, Neuropharmacology, Addiction and Behavior, Uppsala UniversityUppsala, Sweden

**Keywords:** behavior, alcohol use disorder, Y-maze, open field, prefrontal cortex, spontaneous alternations, strain variation, individual differences

## Abstract

Alcohol use disorder (AUD) is a worldwide public health problem and a polygenetic disorder displaying substantial individual variation. This work aimed to study individual differences in behavior and its association to voluntary alcohol intake and subsequent response to naltrexone in a seamless heterogenic group of animals. Thus, by this approach the aim was to more accurately recapitulate the existing heterogeneity within the human population. Male Wistar rats from three different suppliers (Harlan Laboratories B.V., RccHan™:WI; Taconic Farms A/S, HanTac:WH; and Charles River GmbH, Crl:WI) were used to create a heterogenic group for studies of individual differences in behavior, associations to intermittent voluntary alcohol intake and subsequent response to naltrexone. The rats were tested in the open field prior to the Y-maze and then given voluntary intermittent access to alcohol or water in the home cage for 6 weeks, where after, naltrexone in three different doses or saline was administered in a Latin square design over 4 weeks and alcohol intake and preference was measured. However, supplier-dependent differences and concomitant skew subgroup formations, primarily in open field behavior and intermittent alcohol intake, resulted in a shifted focus to instead study voluntary alcohol intake and preference, and the ensuing response to naltrexone in Wistar rats from three different suppliers. The results showed that outbred Wistar rats are diverse with regard to voluntary alcohol intake and preference in a supplier-dependent manner; higher in RccHan™:WI relative to HanTac:WH and Crl:WI. The results also revealed supplier-dependent differences in the effect of naltrexone that were dose- and time-dependent; evident differences in high-drinking RccHan™:WI rats relative to HanTac:WH and Crl:WI rats. Overall these findings render RccHan™:WI rats more suitable for studies of individual differences in voluntary alcohol intake and response to naltrexone and further highlight the inherent heterogeneity of the Wistar strain. The overall results put focus on the importance of thoroughly considering the animals used to aid in study design and for comparison of reported results.

## Introduction

Alcohol use disorder (AUD) is a worldwide public health problem. Despite being a global problem, the approved pharmacotherapy for AUD is restricted to a few substances with varied and limited clinical efficacy (Nutt and Rehm, 2014). Alcohol has a complex and still not fully understood mechanism of action involving many different neurotransmitter systems (Soderpalm and Ericson, 2013; Tabakoff and Hoffman, 2013; Koob, 2014). One system of importance for the reinforcing effects of alcohol and involved in the development of AUD is the endogenous opioid system (Oswald and Wand, 2004; Trigo et al., 2010). In support of this, one of the substances approved for treatment of AUD is the opioid receptor antagonist naltrexone, exerting its effect by blocking the endogenous opioid receptors and thereby the reinforcing effects of alcohol (O'Malley et al., 1992; Volpicelli et al., 1992, 1995). However, a wide variation of treatment effect has been reported and the body of research to deduce the underlying reasons for these variations is numerous. Age at alcohol drinking onset (Kranzler et al., 2003), level of alcohol consumption (Mitchell et al., 2009), classification based on typologies (Leggio et al., 2009), family history of AUD (Garbutt et al., 2014), and genetic alterations (Oliva and Manzanares, 2007; Palmer et al., 2013) have shown to play a key role in the outcome of naltrexone treatment. Moreover, a polymorphism in the μ-opioid receptor gene (Thorsell, 2013) and early life environmental factors (Daoura and Nylander, 2011) impact on the response to naltrexone.

Animal experiments are a necessity to gain a deeper understanding of the physiological, behavioral, and biochemical processes of AUD (Crabbe, 2014), and various methods have been developed to study these processes. Outbred animal strains are generally preferred to more accurately recapitulate the existing heterogeneity within the human population (Stewart and Kalueff, 2015). The Wistar rat is commonly used in AUD research (Clause, 1993) and is the background for many selectively bred alcohol-preferring lines. Although all Wistar rats originate from the Wistar Institute in Philadelphia, generations of breeding at different suppliers have made the Wistar rat diverge, e.g., with regard to voluntary alcohol intake (Palm et al., 2011b; Goepfrich et al., 2013) and differences in endogenous opioid peptide levels (Palm et al., 2012), thus underlining the choice of supplier as an important factor.

Wistar rats from different suppliers have previously been characterized in the multivariate concentric square field™ (MCSF) test (Meyerson et al., 2006) and evident profile differences were observed (Palm et al., 2011a). Moreover, we recently showed that individual differences in risk-assessment behavior were associated with differences in voluntary alcohol intake, while no association between risk-taking behavior and alcohol intake was found (Momeni et al., 2014). Risk-assessment may relate to constructs of decision-making, and impaired decision-making and cognitive dysfunction is associated with excessive drug use (Balogh et al., 2013; Gowin et al., 2013). Although the MCSF test is ethologically founded with the purpose to enable behavioral profiling of the animal within a single test situation to minimize risks of carryover effects (Meyerson et al., 2006), possibilities for studies of specific cognitive function are limited (Karlsson et al., 2009) and yet not validated. Numerous studies have shown the relation of generalized prefrontal cortex dysfunction, impaired cognitive control, and excessive drug use (Goldstein and Volkow, 2011; Bazov et al., 2013), and this area has also been studied to understand mechanisms of compulsiveness and loss of control and the propensity to self-administer drugs (Everitt and Robbins, 2005; George and Koob, 2010).

This work aimed to study individual differences in spontaneous alternations in the Y-maze as indicative of cognitive function (Lalonde, 2002) and its association to voluntary alcohol intake and preference, and the subsequent response to naltrexone. In order to create a seamless heterogenic group of animals, representative of the heterogeneity among the human population (Stewart and Kalueff, 2015) and, likewise, among AUD patients (Hines et al., 2005), Wistar rats from three different suppliers were included. In addition, the open field (OF) test was used to control for potential differences in activity. However, as we will show, the emerging data early on revealed supplier-dependent differences of such magnitude that the aim was shifted to instead focus on voluntary intake of alcohol and the ensuing response to naltrexone. The concluding aim of this paper is thus to behaviorally characterize Wistar rats from three different suppliers in OF activity and Y-maze performance, and thence study intermittent voluntary alcohol intake and preference, and response to naltrexone treatment.

## Material and methods

### Animals

A total of 60 outbred male Wistar rats, 20 rats from three different suppliers, arrived at 8 weeks of age. The suppliers used were Harlan Laboratories B.V., Horst, The Netherlands (RccHan™:WI, referred to as Rcc), Taconic Farms A/S, Ejby, Denmark (HanTac:WH, referred to as Tac) and Charles River, GmbH, Sulzfeld, Germany (Crl:WI, referred to as Crl). The rats were group-housed (2–3 rats/cage) in transparent polysulfone cages (59 × 38 × 20 cm) containing wood chip bedding material and two paper sheets (40 × 60 cm; Cellstoff, Papyrus) as enrichment. The cages were placed in temperature- (21 ± 1°C) and humidity-controlled (50 ± 10%) housing cabinets with a reversed 12 h light/dark cycle (lights off at 07.00 h), and masking background noise. The rats were maintained on standard rat chow (R36; Lantmännen, Kimstad, Sweden) and water *ad libitum*. All animal experiments were approved by the Uppsala Animal Ethical Committee and followed the guidelines of the Swedish Legislation on Animal Experimentation (Animal Welfare Act SFS1998: 56) and the European Communities Council Directive (86/609/EEC).

### Experimental procedure

**Figure 1:**
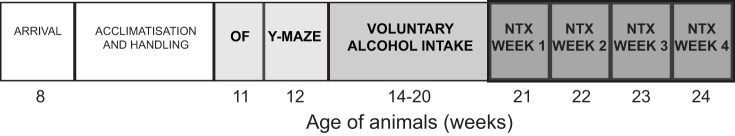
**The experimental outline**. The rats were allowed to acclimatize to the animal facility and the reversed light/dark cycle for 2 weeks and adapted by daily handling. Thereafter, the animals were tested in the open field (OF) followed by the Y-maze and then given intermittent access to alcohol and water in the home cage on 3 consecutive days per week for 6 weeks. Subsequently, over the next 4 weeks naltrexone (NTX) and saline were administered in a Latin square design 30 min prior to alcohol access and intake was measured at 30 min, 2 h, and 24 h.

An overview of the experimental procedure is shown in Figure 1. The rats were allowed to acclimatize and reverse their light/dark cycle for 2 weeks followed by 3 days of adaptation to experimental procedures by handling in a procedure similar to that in previous experiments (Roman et al., 2012). The rats were then tested in the OF followed by the Y-maze. The animals were then single housed and given intermittent access to alcohol solution and water in the home cage for 6 weeks. Subsequently, naltrexone in three different doses or saline was administered in a Latin square design over 4 weeks and alcohol intake was measured.

### The open field test

The OF consists of a black open and illuminated circular arena (diameter 90 cm, 100 lx, Momeni et al., 2014). The animals were started facing the wall and allowed to freely explore for 20 min. In the analysis, the arena is divided into three circular zones, i.e., center (30 cm in diameter), middle circle, and outer circle (each 15 cm wide). Time spent in the inner zone (merged middle circle and center) is used for assessment of risk-taking behavior, thus dividing the animals by central activity vs. thigmotaxis (Momeni et al., 2014).

### The Y-maze test

**Figure 2:**
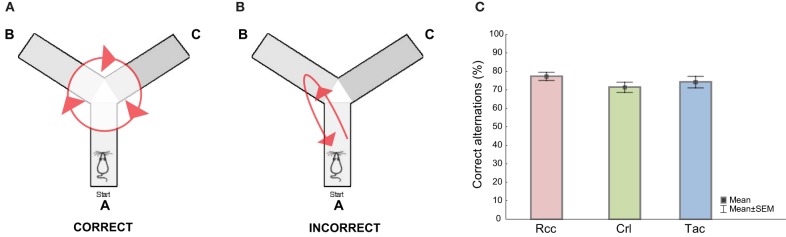
**Schematic illustration showing examples of (A) a correct and (B) an incorrect alternation in the Y-maze test, and (C) the level (%) of correct alternations when comparing Wistar rats from different suppliers, i.e., RccHan™:WI (Rcc), Crl:WI (Crl), and HanTac:WH (Tac)**. Data are presented as mean ± SEM (ANOVA, Fisher's LSD *post-hoc test*).

The Y-maze is constructed of gray, non reflective plastic and consists of three illuminated (100 lx) arms (zone A, B, and C) each 50 cm long, 10 cm wide, and 20 cm high oriented at a 120° angle relative to each other with a central triangular area (mid zone). The animals were started in arm A facing the mid zone and allowed to freely explore for 10 min. Alternation measures the exploratory rotation of the animal and a correct alternation is defined as the animal visiting all arms in a sequential fashion without interruption in its arm choice, thus an alternation of for example A to B to C is correct (Figure 2A) whereas A to B to A is incorrect (Figure 2B), and the percent correct alternations is the number of correct alternations divided by the total number of alternations (Lalonde, 2002).

### Behavioral recordings

The animals were monitored from an adjacent room via a ceiling mounted camera and visits to the defined zones were scored on the premise that both hind legs were within the zone. The latency (LAT, s) of first visiting a zone, frequency (FRQ) of visits, and duration (DUR, s) of time spent in each zone, the number of animals visiting each zone (occurrence, OCC), the total distance (cm) traveled and the mean velocity (cm/s) in the arenas were recorded. In addition, total duration (s) being mobile and immobile, and number of correct and incorrect alternations in the Y-maze were registered. All recordings and analyses were made in the Ethovision system (version XT10 and macro Sequence Analysis toolkit, Noldus Information Technology, Wageningen, the Netherlands).

### Voluntary alcohol intake

The animals were housed individually in cages (42 × 26 × 18 cm) with wooden houses for enrichment, and alcohol was introduced in a previously described intermittent two bottle free-choice paradigm (Momeni and Roman, 2014) slightly modified from Wise (1973), Wayner et al. (1972), and Simms et al. (2008). The animals were given 24 h access to two bottles, tap water and 20% alcohol (v/v), for 3 consecutive days every week. Bottle positions were changed each session to avoid placement preference. Alcohol [diluted from 96% ethanol (Solveco Etanol A 96%; Solveco AB, Rosersberg, Sweden) in tap water] and water were presented at room temperature in 150 ml plastic bottles with minimal spillage ball valve nipples (Scanbur AB, Sollentuna, Sweden). During the days between the alcohol sessions the animals had access to one bottle of water. Alcohol intake was measured at the end of each 24 h session by weighing the bottles and calculated as gram per kilogram. Alcohol preference was calculated as alcohol intake (g) divided by total fluid intake (g). To minimize disturbing factors during the intake measures the cages were changed and animals weighed on a day with access to water only.

### Naltrexone treatment

After 6 weeks of intermittent voluntary alcohol intake, naltrexone treatment was initiated with three doses (0.03, 0.3, or 3 mg/kg) or saline based on previous studies (Daoura and Nylander, 2011; Barson et al., 2013). Treatment was given in a Latin square design so that, at the end, all animals had received all doses and saline. Injections were given prior to the first alcohol session and followed by two wash out sessions each week for 4 consecutive weeks. Naltrexone (Sigma-Aldrich, Schenndorf, Germany) was dissolved in saline and administered subcutaneously (Williams and Broadbridge, 2009) at 1 ml/kg 30 min prior to alcohol access. Alcohol intake, by bottle weighing, was measured at 30 min, 2 h, and 24 h after alcohol access and data presented as Δ0-30, Δ30-2, and Δ0-24, respectively.

### Formation of subgroups

Subgroups were formed using a tertiary split of high, intermediate and low, based on percentage duration in the inner zone of the OF (Momeni et al., 2014), level of correct alternations in the Y-maze, and alcohol intake prior to naltrexone (Momeni and Roman, 2014). The tertiary split into high drinkers (HD), intermediate drinkers (ID) or low drinkers (LD) (Momeni and Roman, 2014) was based on alcohol intake during weeks 1–6 by ranking all animals based on intake per week which was then turned into a sum rank value over the 6 weeks of voluntary intake. The tertiary split was done on all rats, collapsing across supplier origin and then the percentage of rats from each supplier falling into high, intermediate or low groups was calculated.

### Statistical analyses

Body weight data was normally distributed according to the Shapiro-Wilk's W-test; hence the parametric repeated measurement analysis of variance (ANOVA) was used, followed by the Fisher's Least Significant Difference (LSD) *post-hoc* test. Data collected from the behavioral recordings and fluid intake did not show normal distribution according to the Shapiro-Wilk's W-test; hence non-parametric tests were used. For group analysis of behavioral recordings and fluid intake, the non-parametric Kruskal-Wallis test, followed by the Mann-Whitney U-test was used. The Spearman Rank Order Correlation was used for analysis of correlations of descriptive parameters in the OF and Y-maze tests. To compare alcohol and water intake, and alcohol preference over time during the period of naltrexone and saline administrations the Friedman test followed by the Wilcoxon matched pairs test was used. Interaction effects between supplier and effects of naltrexone could not be tested due to the use of non-parametric statistics. Statistica 12.0 (StatSoft Inc., Tulsa, OK) was used for all statistical analyses, including skewness, and differences were considered statistically significant at *p* ≤ 0.05.

## Results

### Body weight

Group-wise analysis of mean body weight weeks 1–6 with alcohol access prior to naltrexone treatment revealed higher body weight in age-matched Crl rats relative to Rcc and Tac animals throughout, whereas no differences were found between Rcc and Tac animals (Supplementary Figure 1).

### The open field test

In general, differences were found comparing Crl rats with Rcc and Tac rats. Specifically all parameters concerning activity and exploratory behavior, and percentage duration in the inner zone were higher in Crl rats (Supplementary Table 1). An overall correlation between the latter parameter and total activity was found (ρ = 0.89, *p* < 0.0001) in all rats, and within the respective group; Rcc (ρ = 0.80, *p* < 0.0001), Crl (ρ = 0.82, *p* < 0.0001), and Tac (ρ = 0.87, *p* < 0.0001).

### The Y-maze test

No differences between groups were found comparing the percentage correct alternations (Figure 2C). Crl rats displayed increased mobility and longer total distance traveled compared to Rcc and Tac, while no differences between Rcc and Tac were found. Analysis of time spent in the arms (zone A, B, or C) revealed no differences and the pattern of alternation was similar for all groups, but Crl spent more time in the mid zone relative to the others (Supplementary Table 2). An overall correlation was found between total distance moved in the OF and the Y-maze (ρ = 0.58, *p* < 0.0001), but without correlations within groups.

### Alcohol intake and preference prior to naltrexone treatment

**Figure 3:**
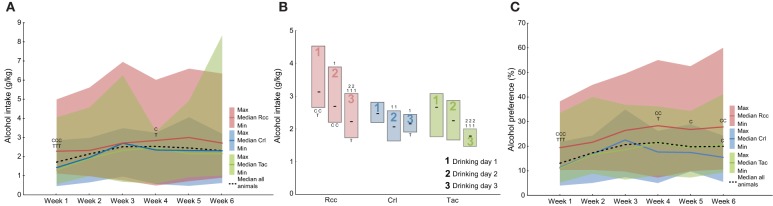
**Weekly voluntary alcohol intake (g/kg, A), average alcohol intake (g/kg) on the 3 days of access (drinking day 1, 2, and 3, B), and alcohol preference (%, C) during the 6 weeks of intermittent access prior to naltrexone treatment in Wistar rats from three different suppliers, i.e., RccHan™:WI (Rcc), Crl:WI (Crl), and HanTac:WH (Tac)**. Data are shown as median with shaded areas indicating min and max **(A,C)**, and median and quartile range **(B)**. ^C^*p* ≤ 0.05, ^CC^*p* < 0.01, ^CCC^*p* < 0.001 compared to Crl rats, ^T^*p* = 0.05 and ^TTT^*p* < 0.001 compared to Tac rats (Mann-Whitney U-test), ^1^*p* = 0.05, ^11^*p* < 0.01, ^111^*p* < 0.001 compared to the intake on drinking day 1 within the respective group, and ^2^*p* = 0.05, ^22^*p* < 0.01, ^222^*p* < 0.001 compared to the intake on drinking day 2 within the respective group (Wilcoxon matched pairs test).

Voluntary alcohol intake was studied using the modified intermittent paradigm with alcohol access for 3 consecutive days per week (Momeni and Roman, 2014). Overall differences were found for week 1 (*H* = 16.12; *p* < 0.001) and 4 (*H* = 6.11; *p* < 0.05) with the highest intake in the Rcc group (Figure 3A). An increase in voluntary alcohol intake over the 6 weeks of access was observed when analyzing all animals (χ^2^ = 44.75; *p* < 0.0001), but also Crl (χ^2^ = 30.54; *p* < 0.0001) and Tac (χ^2^ = 18.89; *p* < 0.05) separately, but not in Rcc. The median and quartile range (QR) of alcohol intake (g/kg) week 6 was: Rcc 2.7 (2.0–4.5), Crl 2.3 (1.8–2.8), and Tac 2.2 (1.7–2.6).

Differences in intake upon regaining alcohol access (drinking day 1 each week) compared to when accustomed (drinking day 3) were assessed by comparing the average intake for drinking days 1, 2, and 3 (Figure 3B). Group-wise comparisons revealed a higher alcohol intake in Rcc on drinking day 1 and 3, with a tendency toward significance (*p* = 0.07) on day 2, compared to Tac, and drinking day 1 and 2 compared to Crl. The intake within groups showed an evident pattern in Rcc with the highest intake on day 1 relative to 2 and 3, whereas in Crl and Tac the pattern was less pronounced but both still had the highest intake on day 1 (Figure 3B).

Overall differences in alcohol preference (%) between groups were found for all weeks except 2 and 3. *Post-hoc* analysis revealed a higher preference in Rcc compared to Tac week 1 and 4, and compared to Crl for all weeks except 2 and 3. At week 6, alcohol preference was higher in Tac relative to Crl (Figure 3C).

### Water and total fluid intake prior to naltrexone treatment

Overall differences were found in water intake for all weeks except 1 and 3, and in total fluid intake for all weeks except 1 and 2 (Supplementary Tables 3A,B). *Post-hoc* analysis revealed a higher water intake in Crl compared to Rcc (weeks 2 and 4–6) and Tac (week 4–6), and a higher total fluid intake in Crl compared to Rcc and Tac (week 4–6 and 3–6, respectively).

### Formation of subgroups

No correlations were found between OF or Y-maze behavior and voluntary alcohol intake (data not shown). The overall skewness for duration in the inner zone of the OF was 1.1 and alcohol load week 6 was 1.6, whereas the level of correct alternations in the Y-maze were more evenly distributed. A skewed pattern was found also within the respective supplier group (Table 1). This finding led to the rejection of the original hypothesis, which was creating subgroups based on an assumed seamless heterogeneity among animals from different suppliers.

**Table 1:** **Formation of subgroups**.

		**Rcc**	**Crl**	**Tac**
**OPEN FIELD**				
	High	30%	60%	10%
	Intermediate	30%	30%	40%
	Low	40%	10%	50%
	Skewness	0.5	0.8	0.8
**Y-MAZE**				
	High	35%	25%	40%
	Intermediate	35%	35%	25%
	Low	30%	40%	35%
	Skewness	0.1	−0.8	−0.1
**ALCOHOL INTAKE**				
	High	65%	15%	20%
	Intermediate	20%	40%	40%
	Low	15%	45%	40%
	Skewness	1.0	0.7	−0.6

*Formation of subgroups based on a tertiary split of percentage duration in the inner zone of the OF, level of correct alternations in the Y-maze and voluntary alcohol intake prior to naltrexone in Wistar rats from three different suppliers, i.e., RccHan−:WI (Rcc), Crl:WI (Crl), and HanTac:WH (Tac). Data are presented as the distribution of animals from a tertiary split into groups of high, intermediate and low for each parameter*.

**Figure 4:**
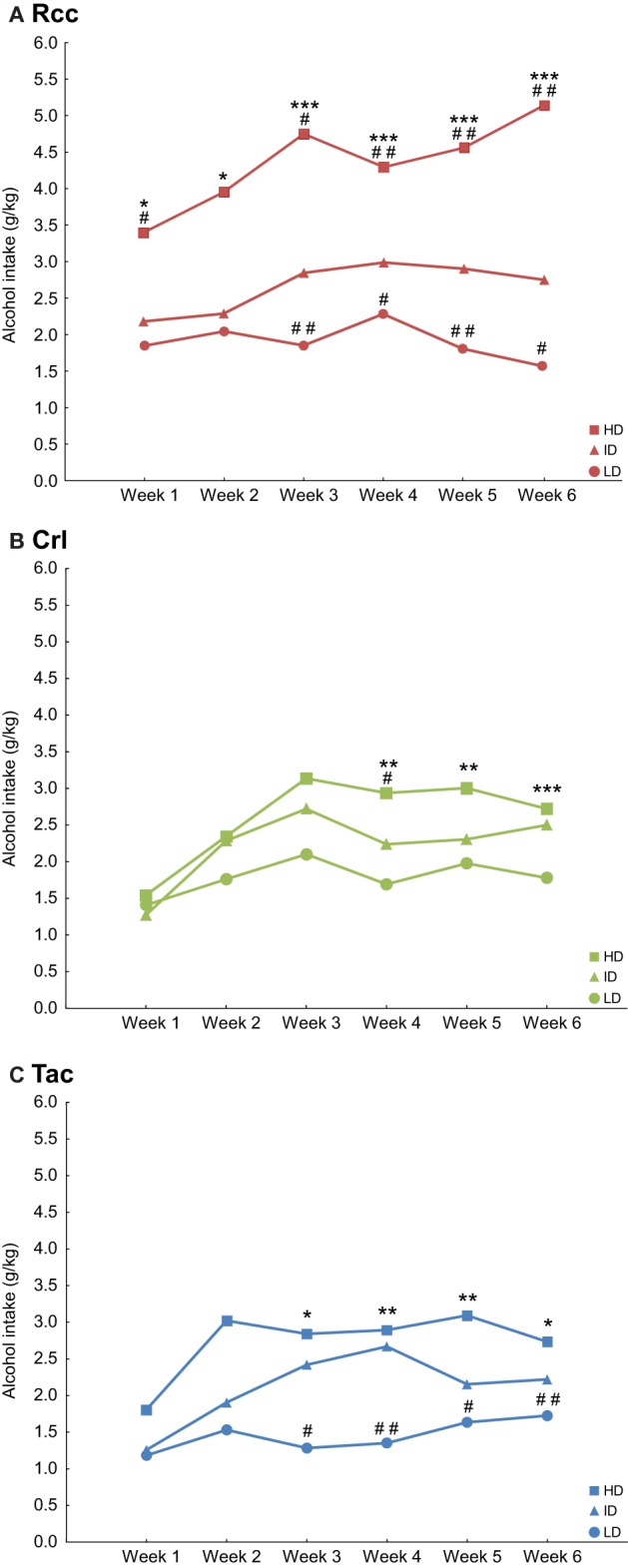
**Voluntary alcohol intake (g/kg) prior to naltrexone in Wistar rats from three different suppliers, i.e., RccHan™:WI (Rcc, A), Crl:WI (Crl, B), and HanTac:WH (Tac, C), divided by a tertiary split into high drinkers (HD), intermediate drinkers (ID) and low drinkers (LD) based on alcohol intake during weeks 1-6**. Data are shown as median. ^*^p ≤ 0.05, ^**^p < 0.01, ^***^p < 0.001 compared to the respective LD group; #*p* ≤ 0.05, ##*p* < 0.01 compared to the respective ID group (Mann-Whitney U-test).

Alcohol intake within each supplier group after a tertiary into HD, ID, and LD subgroups is shown in Figure 4. Rcc showed a large variation in alcohol intake prior to naltrexone treatment as a difference was found between HD and LD for all weeks of access, between HD and ID except for week 2, and ID and LD except weeks 1–2 (Figure 4A). For Crl, a difference was found between HD and LD for weeks 4–6 and between HD and ID for week 4 (Figure 4B). In Tac, a difference was found between HD and LD and between ID and LD for weeks 3–6 (Figure 4C). With regard to alcohol preference during week 6, Rcc-HD displayed higher preference compared to ID and LD but no difference was found comparing Rcc-ID and Rcc-LD. Crl-HD and ID had higher preference compared to Crl-LD, but no difference was found comparing Crl-HD and ID. Similarly, Tac-HD and ID had higher preference compared to Tac-LD, but no difference was found comparing Tac-HD and ID (data not shown).

### The effect of naltrexone on alcohol intake and preference

#### The effect of naltrexone on alcohol intake and preference after 30 min

**Figure 5:**
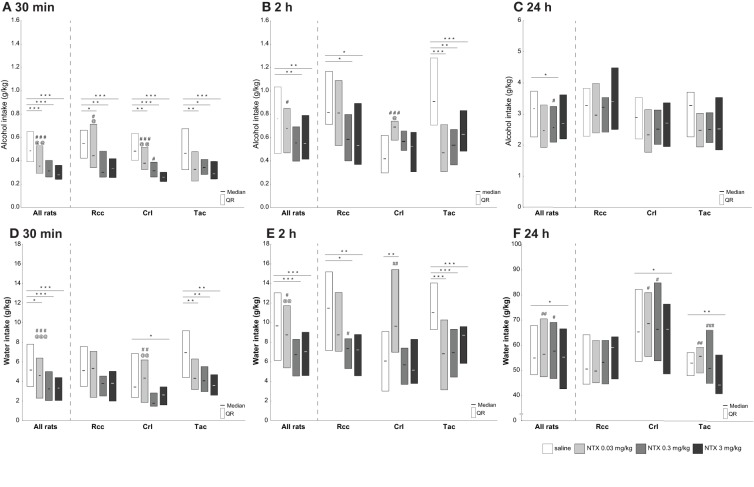
**Voluntary alcohol (A–C) and water (D–F) intake (g/kg) after treatment with saline or naltrexone (0.03, 0.3, or 3 mg/kg) administered s.c. 30 min prior to alcohol access in Wistar rats from three different suppliers, i.e., RccHan™:WI (Rcc), Crl:WI (Crl), and HanTac:WH (Tac)**. Alcohol and water intake was measured at 30 min (**A,D**, respectively), 2 h (**B,E**, respectively), and 24 h (**C,F**, respectively) after access. Data are presented as median and quartile range. ^*^p ≤ 0.05, ^**^p < 0.01, ^***^p < 0.001 compared to saline; @*p* ≤ 0.05, @@*p* < 0.01, @@@*p* < 0.001 compared to naltrexone at 0.3 mg/kg; #*p* ≤ 0.05, ##*p* < 0.01, ###*p* < 0.001 compared to naltrexone at 3 mg/kg (Wilcoxon matched pairs test).

An overall effect of naltrexone on alcohol intake was found in all rats 30 min after alcohol access, and dose-dependent differences were observed when comparing all doses of naltrexone to saline (Figure 5A). Group-wise comparisons showed an overall effect within each group. All three doses of naltrexone reduced alcohol intake compared to saline in Rcc, but no intra-dose differences were observed between the two highest doses. A dose-dependent effect of naltrexone on alcohol intake was seen in Crl, and a general but not dose-dependent effect in Tac (Figure 5A).

When analyzing all rats, no overall effect of naltrexone on alcohol preference was found 30 min after alcohol access, or any differences within each supplier group (data not shown).

#### The effect of naltrexone on alcohol intake and preference after 2 h

An overall effect of naltrexone on alcohol intake was found in all rats 2 h after alcohol access, and naltrexone at 0.3 along with 3 but not 0.03 mg/kg resulted in decreased alcohol intake compared to saline in all rats (Figure 5B). Group-wise comparisons revealed an overall effect of naltrexone on alcohol intake in Rcc and Tac, while a different pattern was observed in Crl. In Rcc, a decrease in intake was found after naltrexone at 0.3 and 3 but not after 0.03 mg/kg. An effect of naltrexone on alcohol intake after all doses compared to saline was found in Tac, but no dose-dependent effect. In Crl, no decrease in alcohol intake compared to saline was found for any dose. Moreover, naltrexone at 0.03 mg/kg resulted in a higher intake compared to 0.3 and 3 mg/kg (Figure 5B).

When analyzing all rats, no overall effect of naltrexone on alcohol preference was found in all rats 2 h after alcohol access, or any differences within each supplier group (data not shown).

#### The effect of naltrexone on alcohol intake and preference after 24 h

After 24 h a minor effect of naltrexone on alcohol intake (Figure 5C) and preference (data not shown) was seen, where naltrexone at 0.3 mg/kg reduced the intake and preference for all rats but there were no differences within each supplier group.

#### The effect of naltrexone on alcohol intake and preference in high-, intermediate-, and low-drinking animals

**Figure 6:**
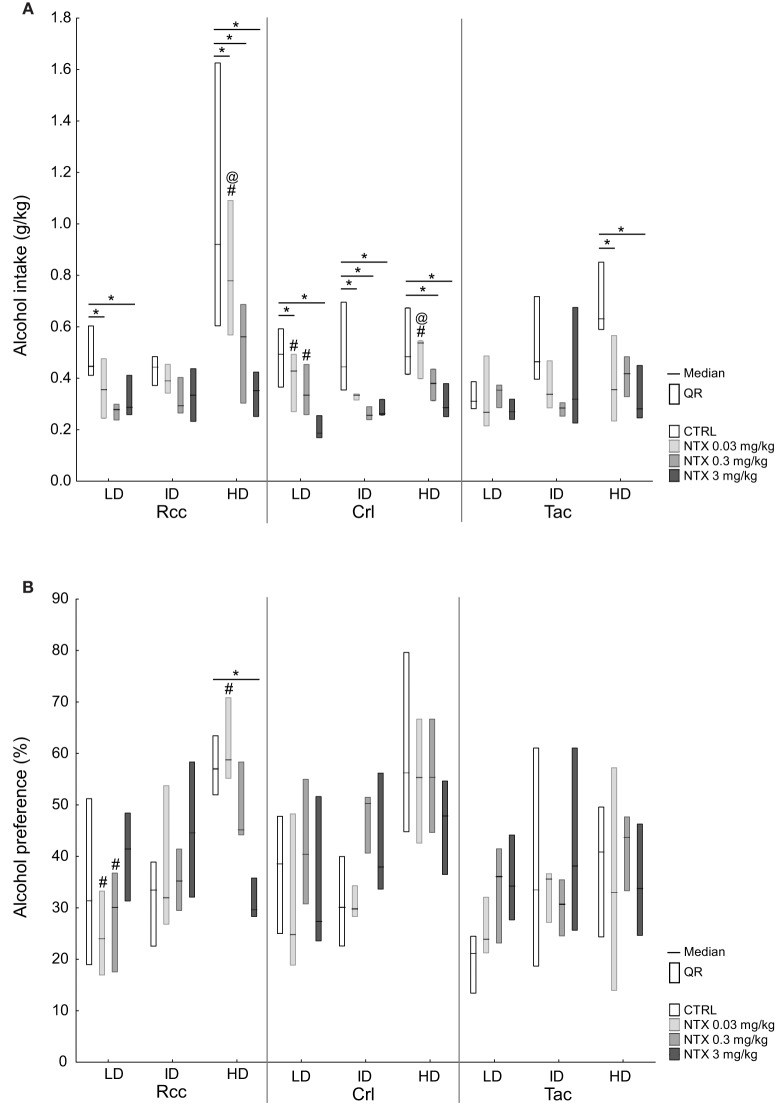
**Voluntary alcohol intake (A) and preference (B) after treatment with saline or naltrexone (0.03, 0.3, or 3 mg/kg) administered s.c. 30 min prior to alcohol access in Wistar rats from three different suppliers, i.e., RccHan™:WI (Rcc), Crl:WI (Crl), and HanTac:WH (Tac), divided by a tertiary split into high drinkers (HD), intermediate drinkers (ID), and low drinkers (LD) based on the alcohol intake during weeks 1–6**. Data are presented as median and quartile range. ^*^p ≤ 0.05 compared to saline; @*p* ≤ 0.05 compared to naltrexone at 0.3 mg/kg; #*p* ≤ 0.05 compared to naltrexone at 3 mg/kg (Wilcoxon matched pairs test).

The response to naltrexone on alcohol intake after 30 min in HD, ID, and LD subgroups within each supplier group is shown in Figure 6A. In Rcc-HD, all three doses of naltrexone reduced alcohol intake compared to saline, but no intra-dose differences were observed between the two highest doses. In Rcc-LD, naltrexone at 0.03 and 0.3 mg/kg resulted in lower alcohol intake, while no effect of naltrexone was found in Rcc-ID. In Crl, all groups responded to naltrexone compared to saline, except at 0.03 mg/kg in Crl-HD, and 0.3 in Crl-LD. In Tac, naltrexone at 0.03 and 3 mg/kg resulted in lower alcohol intake compared to saline in HD, whereas no effect was seen for ID or LD (Figure 6A). No effect of naltrexone on alcohol intake was seen 2 or 24 h after alcohol access (data not shown).

The response to naltrexone on alcohol preference after 30 min in HD, ID, and LD subgroups within each supplier group is shown in Figure 6B. In Rcc-HD, naltrexone at 3 mg/kg reduced alcohol preference compared to saline and naltrexone at 0.03 mg/kg. In Rcc-LD naltrexone at 3 mg/kg resulted in increased preference relative to 0.03 and 0.3 mg/kg; this effect remained also after 2 h. In Rcc-ID, as well as in Crl and Tac subgroups, no effect of naltrexone on alcohol preference was seen after 30 min (Figure 6B) or after 2 h (data not shown). After 24 h some effects on alcohol preference were found. In Crl-ID there was a decrease in preference between saline and naltrexone at 0.03 mg/kg and an increased preference following naltrexone at 3 mg/kg compared to each of the two lower doses. In Rcc-ID there was a decreased preference following naltrexone at 0.3 compared to 3 mg/kg, and decreased in Rcc-LD following naltrexone at 0.03 compared to 3 mg/kg (data not shown).

### The effect of naltrexone on water intake

#### The effect of naltrexone on water intake after 30 min

An overall effect of naltrexone on water intake was found in all animals after 30 min, and group-wise comparisons revealed an overall effect of naltrexone compared to saline in Tac, a less pronounced effect in Crl, and no effect in Rcc. Specifically, naltrexone at 0.3 and 3 mg/kg decreased water intake compared to 0.03 mg/kg in Crl (Figure 5D).

#### The effect of naltrexone on water intake after 2 h

An effect of naltrexone at 0.3 and 3 mg/kg on water intake was found in all animals after 2 h. Group-wise comparisons revealed an overall effect of naltrexone compared to saline in Tac, and a less pronounced effect in Rcc and Crl. In Rcc, a decreased intake compared to saline was found following naltrexone at 0.3 and 3 mg/kg, whereas in Crl an increased water intake after naltrexone at 0.03 mg/kg compared to saline was found (Figure 5E).

#### The effect of naltrexone on water intake after 24 h

A minor, but still significant, effect of naltrexone on water intake was found in all animals after 24 h, and group-wise comparisons revealed an effect of naltrexone compared to saline in Crl and Tac, but not in Rcc. More precisely, water intake decreased in Tac and Crl after naltrexone at 3 mg/kg compared to saline, and compared to 0.03 and 0.3 mg/kg (Figure 5F).

## Discussion

This study aimed to investigate individual differences in behavior, its association to voluntary alcohol intake, and the subsequent response to naltrexone on voluntary alcohol intake in a heterogeneous group of Wistar rats, composed by mixing animals from different suppliers. The rationale for this study design is the recently highlighted limited concordance of animal experiments with subsequent human clinical trials, which urgently warrants progress in the translational value of preclinical data (Howells et al., 2014; Everitt, 2015). Thus, full control and detailed knowledge of the experimental models used are a prerequisite to both understand the pathophysiology of complex human disorders and in the development of novel treatment strategies (Stewart and Kalueff, 2015).

### Behavioral responses, voluntary alcohol intake, and rejection of original hypothesis

Analysis of data (OF, Y-maze, and voluntary alcohol intake) revealed supplier-dependent differences and concomitant skew subgroup formations of such magnitude that the direction of the study needed to be changed. The differences, primarily in OF performance and voluntary alcohol intake, were of a supplier-dependent origin rather than equally distributed in the heterogeneous group (Table 1). Subgroup formations based on OF behavior were skewed as the Crl animals were more active, in line with previous studies in our group (Palm et al., 2011a), and had the highest percentage duration in the inner zone, i.e., risk-taking behavior (Momeni et al., 2014), compared to Tac and Rcc rats. No association between risk-taking behavior in the OF and voluntary alcohol intake was found, in line with previous results (Momeni et al., 2014), and subgroup formations based on the analysis of correct alternations in the Y-maze were more evenly distributed. The MCSF test has previously been used for assessment of cognitive performance (Karlsson et al., 2009), however, detailed measures of this need further validation. Compared to other tests used for assessment of cognitive function spontaneous alternations in the Y-maze is based on an innate response and does not require extensive training of animals, which is advantageous in the study design used. Signs of neuroadaptation and changes in cognitive ability, measured as choice behavior in the Y-maze and spontaneous alternations, have shown to be influenced by prefrontal cortex (Pickering et al., 2015) rather than hippocampal function (Lalonde, 2002). Intermittent exposure to alcohol resulted in impaired Y-maze performance as assessed by spontaneous alternations (Gotesson et al., 2012), whereas this study was designed to assess a potential association between individual differences in spontaneous alternation patterns in the Y-maze and voluntary alcohol intake, which could not be found (data not shown). In addition, subgroup formation by a tertiary split based on voluntary alcohol intake prior to naltrexone treatment revealed a skewed distribution as the Rcc animals had notably higher alcohol intake compared to Tac and Crl rats, in agreement with Palm et al. (2011b) and Goepfrich et al. (2013). In summary, the primary aim of creating a seamless heterogenic group of outbred animals using Wistar rats from different suppliers for studies of individual predisposed differences in behavior and associations with voluntary alcohol intake was abandoned. Therefore, the aim was shifted to focus on voluntary alcohol intake and the ensuing response to naltrexone in outbred Wistar rats from different suppliers.

### Alcohol intake and preference prior to naltrexone treatment

Primarily, data presented herein show that outbred Wistar rats differ with regard to voluntary alcohol intake in a supplier-dependent manner. The Rcc group displayed the highest alcohol intake and preference, which also is supported by previous studies (Palm et al., 2011b; Goepfrich et al., 2013), and the level of voluntary alcohol intake in the Rcc group, using the same intermittent two-bottle model, is also replicated from a previous study in our lab (Momeni and Roman, 2014). Notably, the alcohol intake patterns differed somewhat between the Wistar groups. Rcc rats exhibited a stabile intake that peaked more or less upon initial access, which has been shown previously (Palm et al., 2011b; Goepfrich et al., 2013; Momeni and Roman, 2014), while Crl and Tac rats showed an initial increase followed by stabilization (Palm et al., 2011b). The lack of a pronounced escalation in intake was similar to previous reports on intermittent access paradigms (Adermark et al., 2011; Palm et al., 2011b; Suchankova et al., 2013; Momeni and Roman, 2014; Momeni et al., 2014), but in contrast to others (Carnicella et al., 2014).

Comparing intake on the days of alcohol access, i.e., drinking days, the Rcc group displayed increased intake on drinking day 1 relative to day 2 or day 3, which implies an alcohol deprivation effect as previously observed (Momeni and Roman, 2014), while not as distinct in the Tac and Crl groups.

Pronounced subgroup-dependent differences were found upon a tertiary split of the respective Wistar groups into HD, ID, and LD based on voluntary alcohol intake week 1–6 prior to naltrexone treatment. Individual differences were particularly apparent in Rcc rats, thus confirming previous findings (Momeni and Roman, 2014) and in agreement with others (Steensland et al., 2012; Fredriksson et al., 2015). Rcc-HD animals differed from all other groups by a consistently higher intake during all 6 weeks, whereas Rcc-ID/LD animals more closely resembled Crl and Tac rats.

Condensed, these data in our view render the Rcc rats more suitable for studies of voluntary alcohol intake, especially regarding individual differences, than Crl and Tac rats.

### The effect of naltrexone on alcohol intake and preference

The doses of naltrexone used were based on previous studies (Daoura and Nylander, 2011; Barson et al., 2013) and enabled a detailed assessment of dose-dependent responses in the different groups. Examining the data as one heterogeneous group, i.e., in all rats, a dose-dependent response to naltrexone was revealed. The effect of naltrexone compared to saline was seen following all doses as early as 30 min after alcohol access, and still evident in the highest doses (0.3 or 3 mg/kg) after 2 h. This is in agreement with others using similar doses of naltrexone at 30 min (Simms et al., 2008; Daoura and Nylander, 2011) and 2 h (Daoura and Nylander, 2011) after alcohol access.

Supplier-dependent differences in the effect of naltrexone were observed, as the effect was discontinued in Crl rats and only observed for the two highest doses in Rcc rats at 2 h. An overall effect was seen for all doses in Tac rats at 2 h, and the effect had ceased in all groups 24 h after access. It can not be excluded that the lack of dose dependent effects of naltrexone in the Tac group is due to a floor effect (Cichelli and Lewis, 2002). Others report that naltrexone at 0.02 mg/kg decreased alcohol intake after 2 h in a subgroup of Sprague-Dawley rats displaying high novelty-induced activity (Barson et al., 2013), which may resemble the Crl rats studied here with high OF activity and loss of naltrexone effect at 2 h. In line with the present results, naltrexone at 1 and 2 mg/kg decreased alcohol intake 4 h after alcohol access in Rcc rats (Steensland et al., 2012; Fredriksson et al., 2015).

Assessing naltrexone effects at 30 min in HD, ID and LD groups reveled that Rcc-HD animals stand out with a combined dose-dependent decrease in alcohol intake and a decrease in alcohol preference following the highest dose, not evident in the Rcc-ID or LD animals or any other supplier subgroup. The effect in the Rcc-HD group can be attributed to increased intake in this subgroup, as previous studies describe a more striking effect of naltrexone in individuals with a high level of alcohol intake (Mitchell et al., 2009). Also, naltrexone at 1 and 2 mg/kg reduced alcohol intake in Rcc rats with a similar intermittent voluntary alcohol intake as herein (Steensland et al., 2012; Fredriksson et al., 2015). Furthermore, naltrexone was more effective in reducing voluntary alcohol intake in animals subjected to rearing conditions simulating an unsafe early life environment and concomitant higher adult voluntary alcohol intake (Nylander and Roman, 2013) relative to a simulated protective environment (Daoura and Nylander, 2011).

In addition to the effects of naltrexone on alcohol intake, Rcc-HD animals stand out with a decrease in alcohol preference following the highest dose, which further supports its efficacy in certain subgroups (Mitchell et al., 2009) and adds to the results following administration of the opioid antagonist naloxone (Cichelli and Lewis, 2002). In contrast, in the Rcc-LD group naltrexone at 3 mg/kg resulted in increased preference. This finding is in agreement with a previous study in which naltrexone administration resulted in increased preference for 20% alcohol in animals reared in a more beneficial early life environment with concomitant lower adult voluntary alcohol intake (Daoura and Nylander, 2011; Nylander and Roman, 2013) and consistent with an increased alcohol intake after naltrexone in individuals with no family history of AUD (Krishnan-Sarin et al., 2007). No effect of naltrexone on preference was seen in Rcc-ID animals or any other supplier subgroup. Thus, the impact of naltrexone on alcohol preference in addition to its effects on water intake (see below) renders some of the effects questionable; this may partly be due to a floor effect as previously proposed (Cichelli and Lewis, 2002) or explained by the low/moderate level of alcohol intake in the Rcc-ID group as well as the Crl and Tac subgroups.

### The effect of naltrexone on water intake

In all rats as a heterogeneous group, a dose-dependent response to naltrexone on water intake was found at 30 min, which also was evident for the two highest doses at 2 h. Moreover, there were supplier-dependent differences in the effect of naltrexone on water intake, which were dose- and time-dependent. In Tac rats there was a general effect of naltrexone on water intake at 30 min and 2 h, whereas in Rcc rats there was no effect at 30 min but of the two highest doses at 2 h. In contrast, the Crl rats had an unclear effect at 30 min and the lowest dose resulted in an increased water intake at 2 h. Previous reporting on the effects of naltrexone on water intake is sparse, and findings in the literature are also inconsistent. Studies report on its particular effect on alcohol intake and absent effect on water intake (Daoura and Nylander, 2011; Barson et al., 2013), attenuated fluid intake in general, including water (Lang et al., 1982; Escher and Mittleman, 2006; Simms et al., 2008; Steensland et al., 2012), and some do not present data on water intake, thereby making the understanding of exact mechanisms and effects of naltrexone difficult to dissect. It is well-established that the opioid system is also involved in regulating appetite control and general ingestion (Reid, 1985; Cichelli and Lewis, 2002), which can in part explain the non-alcohol specific effects of naltrexone.

### General discussion

Discussions about the most appropriate experimental design centers on the pivotal question of maximizing or minimizing variance by using outbred or inbred animals and the strict use of constant controlled conditions. From a population validity point of view it has been argued that the use of heterogeneous outbred animals is advantageous for translatability into human disorders, especially upon division into subgroups for studies of individual differences, responders and non-responders etc. (Stewart and Kalueff, 2015). We here attempted to create a seamless heterogenic group by mixing Wistar rats from different suppliers to be divided into subgroups based on individual differences in behavior, voluntary alcohol intake, and subsequent response to pharmacologic treatment. However, due to the skewed distribution of animals related to supplier origin, this was impossible. On the contrary, by comparing Wistar rats from the different suppliers we were able to replicate data from previous studies (Daoura and Nylander, 2011; Palm et al., 2011b; Goepfrich et al., 2013; Momeni and Roman, 2014; Momeni et al., 2014). Most researchers are aware that differences between rat strains and lines from different suppliers exist. However, there are surprisingly few articles on this topic, especially considering how important the awareness of such differences is when selecting the appropriate animals for testing experimental hypotheses as well as for comparison of results between studies. In recent years, systematic studies on this topic have been conducted (Palm et al., 2011a,b, 2012; Goepfrich et al., 2013) that hopefully will increase future interest in this field of research.

Considering alcohol's complex and yet not fully understood mechanism of action (Soderpalm and Ericson, 2013; Tabakoff and Hoffman, 2013; Koob, 2014) it is difficult to speculate about underlying causes for the results presented herein. Antecedent studies on Wistar rats from different suppliers revealed differences in opioid peptide levels, both basal and alcohol-induced, which also correlated to voluntary alcohol intake (Palm et al., 2012). As the opioid system is highly relevant for regulating alcohol intake (Oswald and Wand, 2004; Trigo et al., 2010) and the concomitant effect of naltrexone (Volpicelli et al., 1992; Thorsell, 2013), individual differences in the opioid system (Oswald and Wand, 2004) may play a role. The presented results on voluntary alcohol intake and preference reflect the current literature, where wide variations in intake are reported among drinking paradigms (Crabbe et al., 2010; Becker, 2013; Carnicella et al., 2014), and pinpoints important factors for consideration.

Generally, the findings herein indicate that the heterogeneity of Wistar rats can offer advantages to translational research. Although AUD is diagnosed by diagnostic criteria, which evaluate several aspects of the disorder, this patient group displays great heterogeneity (Hines et al., 2005; Leggio et al., 2009). For instance, naltrexone shows a good overall effect in humans but when studying the population in subgroups there are obvious differences (Thorsell, 2013). The confirmation of a wide heterogeneity within the Wistar rat and the possibility for detailed studies of subgroups adds pieces of understanding to the puzzle of different pharmacological responders.

## Conclusion

In search of individual differences in this study, multiple methodologies were used; behavioral assessment, voluntary alcohol consumption, and pharmacological intervention. The major finding is that the voluntary alcohol consumption and the concomitant response to naltrexone are different in commonly used Wistar rats from different suppliers. In addition, there are differences in the behavior of animals from the different suppliers in the OF, and to a less extent in the Y-maze, that adds to the complexity of individual differences vs. supplier-dependent effects. The condensed data, in our view, render the Rcc Wistar rats more suitable for studies of individual differences in voluntary alcohol intake and preference, and response to pharmacological treatment; this due to an increased alcohol intake, similar to an alcohol deprivation effect, when regaining alcohol using intermittent access, and the presence of a distinguishable high-drinking subpopulation with increasing alcohol intake over time and pronounced effects of naltrexone on alcohol intake and preference. Finally, it is the ambition of the authors that the results presented herein will aid in understanding the sometimes large variation and discrepancy reported, and that it can be used henceforth to aid in study design and for comparison of reported results. The overall results put focus on the importance of thoroughly considering the animals used in future research and acknowledge the fact that “a Wistar rat is not just any Wistar rat.”

## Author contributions

Study PI: ER. Conceived and designed the experiment and interpreted the results: SM, LS, and ER. Performed the experiments: SM and LS. Analyzed the data: SM, LS and ER. Drafted the manuscript: SM. Revised and approved the final version: SM, LS, and ER.

## Funding and disclosure

Financial support from the Alcohol Research Council of the Swedish Alcohol Retailing Monopoly, and the Facias and Åke Wiberg Foundations (ER) is gratefully acknowledged. The authors declare no conflict of interest.

### Conflict of interest statement

The authors declare that the research was conducted in the absence of any commercial or financial relationships that could be construed as a potential conflict of interest.
