# Supplementary material for: Supplier-dependent differences in intermittent voluntary alcohol intake and response to naltrexone in Wistar rats
Source: Front Neurosci. 2015 Nov 5;9:424. doi: 10.3389/fnins.2015.00424 (PMC4633506; doi:10.3389/fnins.2015.00424)
Supplement: Supplementary file 1 [file Supplementary_material.PDF]

Supplementary material

Supplier-dependent differences in intermittent voluntary alcohol intake and response to naltrexone in Wistar rats

Shima Momeni*, Lova Segerström and Erika Roman

Department of Pharmaceutical Biosciences; Neuropharmacology, Addiction and Behavior, Uppsala University, Uppsala, Sweden

*Corresponding Author
Department of Pharmaceutical Biosciences; Neuropharmacology, Addiction and Behaviour, Uppsala University, P.O. Box 591, SE-751 24 Uppsala, Sweden
Telephone: +46-18-471 4620
E-mail: Shima.Momeni@farmbio.uu.se

Supplementary Figure 1.

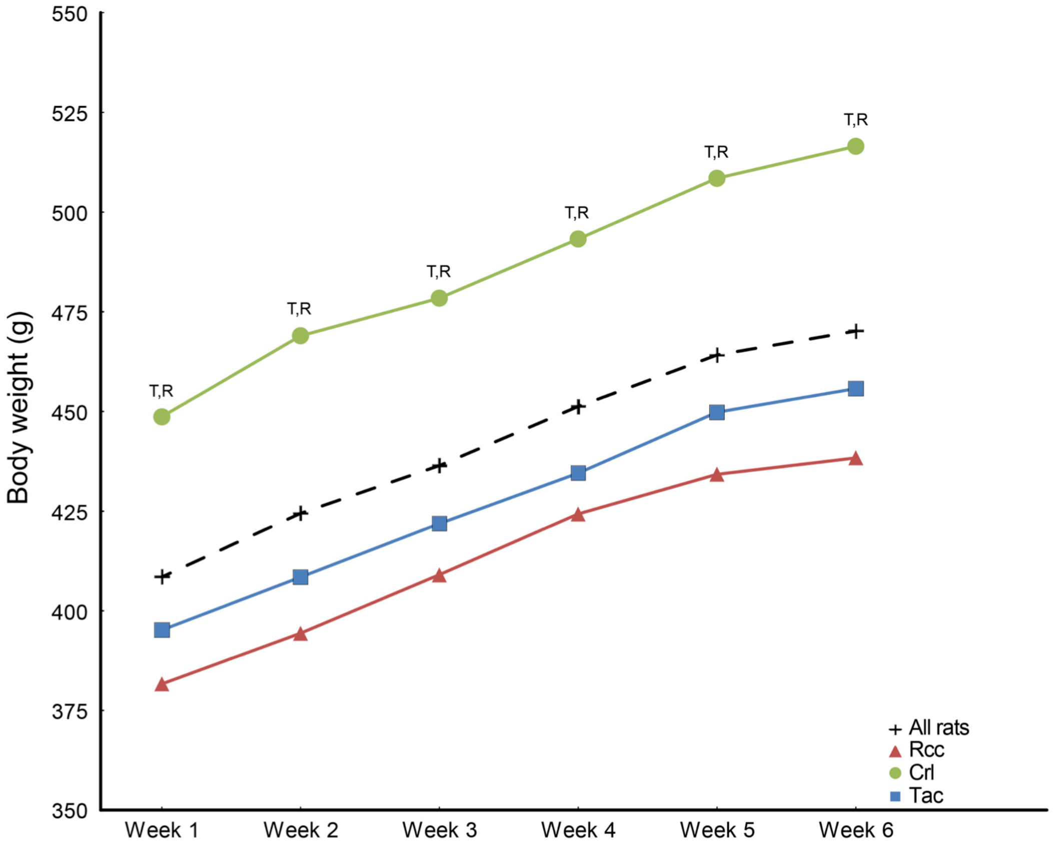

Supplementary Figure 1.
Body weight (g) during the six weeks of voluntary intermittent access to alcohol prior to naltrexone treatment in Wistar rats from different suppliers, i.e. RccHan^{TM}:WI (Rcc), Crl:WI (Crl) and HanTac:WH (Tac). Data are shown as mean. $^{R}p \leq 0.05$ compared to Rcc rats, $^{T}p \leq 0.05$ compared to Tac rats (ANOVA followed by Fisher's Least Significant Difference).

Supplementary Table 1. Results from the open field test

Behavioral parameters during the 20-minute OF test in Wistar rats from three different suppliers, i.e. RccHan™:WI (Rcc), Crl:WI (Crl) and HanTac:WH (Tac). Data are presented as median and quartile range (QR). $^{T}p \leq 0.05$ compared to Tac rats and $^{R}p \leq 0.05$ compared to Rcc rats (Mann-Whitney U-test). Abbreviations: DUR, duration (s); DUR/FRQ, duration (s) per visit; %DUR, percentage duration in relation to the total test time; FRQ, frequency of visits; LAT, latency (s); TOTACT, total activity, i.e. the sum of all frequencies; TOT, total. Occurrence (OCC) is shown for the latency (LAT) measure, as this parameter was regarded as a missing value for animals that did not visit the particular zone.

	All rats		Rcc		Crl		Tac		Kruskal-Wallis test
	Median	QR	Median	QR	Median	QR	Median	QR	
**Outer circle**									
FRQ	80.5	70.5	89.0	77.0	110.5 [R,T]	69.0	46.5	42.0	H=16.5; p<0.001
DUR	1107.3	103.7	1120.3	111.5	1071.2 [R,T]	89.5	1155.3	59.5	H=16.8; p<0.001
DUR/FRQ	13.6	18.6	12.1	26.2	9.9 [R,T]	6.3	24.2	21.2	H=17.3; p<0.001
%DUR	92.3	8.6	93.4	9.3	89.3 [R,T]	7.5	96.3	5.0	H=16.8; p<0.001
**Middle circle**									
LAT	0.4	34.4	0.1	4.1	0.3	27.2	0.04	0.6	n.s.
FRQ	78.0	76.5	68.5	76.0	126.5 [R,T]	73.5	43.0	41.0	H=21.2; p<0.0001
DUR	80.0	77.0	64.0	88.2	113.3 [R,T]	78.6	40.5	61.4	H=14.3; p<0.001
DUR/FRQ	1.0	0.5	1.1	0.4	0.9	0.3	0.9	0.8	n.s.
%DUR	6.7	6.4	5.3	7.4	9.5 [R,T]	6.5	3.4	5.1	H=14.3; p<0.001
**Center**									
LAT	141.7	188.0	191.2	161.0	78.2 [R,T]	87.8	264.8	399.6	H=18.7; p<0.001
FRQ	9.0	18.0	6.5	11.5	20.0 [R,T]	14.0	4.5	7.0	H=20.6; p<0.0001
DUR	8.6	16.3	4.7	10.5	18.3 [R,T]	20.6	3.5	5.9	H=17.9; p<0.001
DUR/FRQ	0.9	0.9	0.7	1.2	0.9	0.2	0.7	1.4	n.s.
%DUR	0.7	1.4	0.4	0.9	1.5 [R,T]	1.7	0.3	0.5	H=17.9; p<0.001
OCC LAT	60 / 60		15 / 20		20 / 20		15 / 20		
**Inner zone**									
DUR	92.2	104.5	78.5	112.4	127.6 [R,T]	88.7	42.2	60.1	H=16.9; p<0.001
%DUR	7.7	8.7	6.5	9.4	10.6 [R,T]	7.4	3.5	5.0	H=16.9; p<0.001
**Others**									
TOTAC	178.5	149.5	175.0	158.0	255.5 [R,T]	142.5	101.0	74.0	H=20.3; p<0.0001
Rearing	35.5	39.0	29.5	14.5	75.5 [R,T]	24.0	26.5	18.5	H=35.3; p<0.0001
Grooming	4.0	3.0	3.5	3.0	4.0	4.0	5.0	5.0	n.s.
DISTANCE TOT	15909.7	4396.4	14035.7	2369.6	19191.1 [R,T]	2260.7	14453.4	3588.0	H=33.9; p<0.0001
VELOCITY TOT	13.3	3.7	11.7	2.0	16.0 [R,T]	1.9	12.0	3.0	H=33.9; p<0.0001

Supplementary Table 2. Results from the Y-maze test

Behavioral parameters during the 10-minute Y-maze test in Wistar rats from three different suppliers, i.e. RccHan^{TM}:WI (Rcc), Crl:WI (Crl) and HanTac:WH (Tac). Data are presented as median and quartile range (QR). $^{T}p \leq 0.05$ compared to Tac rats and $^{R}p \leq 0.05$ compared to Rcc rats (Mann-Whitney U-test). Abbreviations: DUR, duration (s); DUR/FRQ, duration (s) per visit; %DUR, percentage duration in relation to the total test time; FRQ, frequency of visits; LAT, latency (s); TOTACT, total activity, i.e. the sum of all frequencies; TOT, total.

	All rats		Rcc		Crl		Tac		Kruskal-Wallis test
	**Median**	**QR**	**Median**	**QR**	**Median**	**QR**	**Median**	**QR**	
**Zone A**									
FRQ	11.0	5.0	11.0	4.0	14.0^{R,T}	4.5	10.0	3.0	H=13.5; p<0.05
DUR	226.9	49.3	240.2	53.3	211.8	47.1	229.0	53.2	n.s.
DUR/FRQ	19.9	9.8	19.9	14.2	15.7^{R,T}	5.8	23.0	4.8	H=17.9; p<0.001
%DUR	37.8	8.3	40.1	8.9	35.3	7.8	38.2	8.8	n.s.
**Zone B**									
LAT	40.6	38.7	49.6	22.8	20.6^{R,T}	27.7	44.2	30.0	H=8.0; p<0.05
FRQ	9.0	4.5	9.0	3.0	11.5^{R,T}	5.0	8.0	4.0	H=14.8; p<0.001
DUR	148.5	38.7	141.3	47.1	161.2	22.4	136.5	41.8	n.s.
DUR/FRQ	15.1	7.3	16.8	6.7	13.1^{R,T}	3.2	16.3	7.6	H=11.7; p<0.001
%DUR	24.7	6.4	23.5	7.8	26.9	3.8	22.8	7.1	n.s.
**Zone C**									
LAT	32.6	57.1	26.3	59.8	21.7^{T}	36.5	64.3	52.8	H=8.3; p<0.05
FRQ	10.0	4.5	9.0	3.5	12.0^{R,T}	2.5	8.0	4.0	H=19.5; p<0.05
DUR	161.7	41.8	170.1	35.6	161.0	45.6	150.6	37.6	n.s.
DUR/FRQ	15.4	6.8	17.4	9.3	13.3^{R,T}	4.4	17.4	9.9	H=14.8; p<0.001
%DUR	27.0	7.0	28.4	5.9	26.9	7.6	25.2	6.3	n.s.
**Mid zone**									
LAT	19.3	25.1	26.5	25.4	12.2^{R,T}	11.9	22.9	26.8	H=18.1; p<0.001
FRQ	28.0	11.0	26.5	6.5	39.0^{R,T}	10.0	25.0	6.0	H=28.9; p<0.0001
DUR	39.7	19.5	33.2	16.2	47.7^{R,T}	17.9	40.2	25.5	H=7.5; p<0.05
DUR/FRQ	1.3	0.7	1.2	0.6	1.2	0.4	1.7	1.1	n.s.
%DUR	6.6	3.2	5.5	2.7	8.0^{R}	3.0	6.7	4.3	H=7.4; p<0.05
**Others**									
MOBILITY	121.6	48.0	110.3	33.3	140.9^{R,T}	21.2	102.5	47.0	H=13.9; p<0.001
IMMOBILITY	477.1	48.8	488.6	32.1	456.2^{R,T}	21.8	496.5	49.4	H=13.9; p<0.001
DISTANCE TOT	2486.3	723.1	2365.0	529.7	2915.9^{R,T}	652.1	2140.0	681.5	H=25.5; p<0.0001
VELOCITY TOT	4.2	1.2	4.0	0.9	4.9^{R,T}	1.1	3.6	1.1	H=25.4; p<0.0001

Supplementary Table 3. Water and total fluid intake

Weekly water (A) and total fluid (B) intake in Wistar rats from three different suppliers, i.e. RccHan^{TM}:WI (Rcc), Crl:WI (Crl) and HanTac:WH (Tac), during six weeks of intermittent alcohol intake prior to naltrexone treatment. Data of intake (g/kg) are presented as median and min-max. $^{T}p \le 0.05$ compared to Tac rats and $^{R}p \le 0.05$ compared to Rcc rats (Mann-Whitney U-test).

A. Water intake

	All rats		Rcc		Crl		Tac		Kruskal-Wallis test
	Median	Min-max	Median	Min-max	Median	Min-max	Median	Min-max	
**Week 1**	65.5	33.8-123.2	57.2	42.5-103.0	72.2	33.8-123.2	65.8	52.0-108.8	n.s.
**Week 2**	62.4	31.9-122.3	57.2	31.9-93.8	72.5^{R}	46.5-122.3	57.9	45.3-96.2	H=7.7; p<0.05
**Week 3**	58.9	29.8-136.0	57.1	29.8-82.1	67.8	38.7-136.0	59.7	39.3-112.0	n.s.
**Week 4**	60.7	25.8-116.4	53.1^{T}	25.8-76.7	71.3^{R,T}	46.7-116.4	54.7	38.4-94.6	H=13.5; p<0.05
**Week 5**	59.2	27.8-121.9	51.9^{T}	27.8-82.2	68.0^{R,T}	52.2-92.4	56.0	42.9-121.9	H=12.8; p<0.001
**Week 6**	56.6	21.9-125.4	51.7^{T}	22.0-77.1	76.6^{R,T}	52.0-125.4	48.6	35.5-81.3	H=19.5; p<0.001

B. Total fluid intake

	All rats		Rcc		Crl		Tac		Kruskal-Wallis test
	Median	Min-max	Median	Min-max	Median	Min-max	Median	Min-max	
**Week 1**	78.4	48.2-133.5	72.2	60.9-115.3	86.8	48.2-133.5	78.5	59.9-114.7	n.s.
**Week 2**	78.6	56.4-140.2	75.7	56.4-104.6	85.7	57.5-140.2	75.1	61.6-106.4	n.s.
**Week 3**	68.3	37.3-150.4	78.3^{T}	54.2-97.6	88.8^{T}	54.3-150.4	48.5	37.3-88.5	H=30.4; p<0.0001
**Week 4**	76.7	53.2-142.6	74.0	53.2-129.9	86.9^{R,T}	62.6-142.6	67.0	58.5-107.4	H=10.7; p<0.05
**Week 5**	76.2	52.2-131.4	68.8	52.2-99.7	84.3^{R,T}	70.3-121.9	68.3	57.6-131.4	H=9.8; p<0.05
**Week 6**	73.2	49.4-147.1	72.4	49.4-98.8	92.8^{R,T}	65.0-147.1	63.1	51.2-113.1	H=13.4; p<0.001
